# Influence of Sodium Bicarbonate on Wall Teichoic Acid Synthesis and β-Lactam Sensitization in NaHCO_3_-Responsive and Nonresponsive Methicillin-Resistant Staphylococcus aureus

**DOI:** 10.1128/spectrum.03422-22

**Published:** 2022-11-15

**Authors:** Selvi C. Ersoy, Barbara Gonçalves, Gonçalo Cavaco, Adhar C. Manna, Rita G. Sobral, Cynthia C. Nast, Richard A. Proctor, Henry F. Chambers, Ambrose Cheung, Arnold S. Bayer

**Affiliations:** a The Lundquist Institute, Torrance, California, USA; b Laboratory of Molecular Microbiology of Bacterial Pathogens, UCIBIO, Applied Molecular Biosciences Unit, Department of Life Sciences, Nova School of Science and Technology, Universidade Nova de Lisboa, Caparica, Portugal; c Associate Laboratory i4HB, Institute for Health and Bioeconomy, Nova School of Science and Technology, Universidade Nova de Lisboa, Caparica, Portugal; d Department of Microbiology & Immunology, Geisel School of Medicine at Dartmouth, Hanover, New Hampshire, USA; e Cedars-Sinai Medical Center, Los Angeles, California, USA; f Geffen School of Medicine at UCLA, Los Angeles, California, USA; g Department of Medicine, University of Wisconsin School of Medicine and Public Health, Madison, Wisconsin, USA; h Department of Medical Microbiology/Immunology, University of Wisconsin School of Medicine and Public Health, Madison, Wisconsin, USA; i UCSF School of Medicine, San Francisco, California, USA; Riverside University Health System, Medical Center -University of California

**Keywords:** methicillin-resistant *Staphylococcus aureus* (MRSA), sodium bicarbonate, penicillin-binding proteins (PBPs), peptidoglycan (PG), wall teichoic acid (WTA), β-lactams, methicillin resistance

## Abstract

Methicillin-resistant Staphylococcus aureus (MRSA) strains pose major treatment challenges due to their innate resistance to most β-lactams under standard *in vitro* antimicrobial susceptibility testing conditions. A novel phenotype among MRSA, termed “NaHCO_3_ responsiveness,” where certain strains display increased susceptibility to β-lactams in the presence of NaHCO_3_, has been identified among a relatively large proportion of MRSA isolates. One underlying mechanism of NaHCO_3_ responsiveness appears to be related to decreased expression and altered functionality of several genes and proteins involved in cell wall synthesis and maturation. Here, we studied the impact of NaHCO_3_ on wall teichoic acid (WTA) synthesis, a process intimately linked to peptidoglycan (PG) synthesis and functionality, in NaHCO_3_-responsive versus -nonresponsive MRSA isolates. NaHCO_3_ sensitized responsive MRSA strains to cefuroxime, a specific penicillin-binding protein 2 (PBP2)-inhibitory β-lactam known to synergize with early WTA synthesis inhibitors (e.g., ticlopidine). Combining cefuroxime with ticlopidine with or without NaHCO_3_ suggested that these latter two agents target the same step in WTA synthesis. Further, NaHCO_3_ decreased the abundance and molecular weight of WTA only in responsive strains. Additionally, NaHCO_3_ stimulated increased autolysis and aberrant cell division in responsive strains, two phenotypes associated with disruption of WTA synthesis. Of note, studies of key genes involved in the WTA biosynthetic pathway (e.g., *tarO*, *tarG*, *dltA*, and *fmtA*) indicated that the inhibitory impact of NaHCO_3_ on WTA biosynthesis in responsive strains likely occurred posttranslationally.

**IMPORTANCE** MRSA is generally viewed as resistant to standard β-lactam antibiotics. However, a NaHCO_3_-responsive phenotype is observed in a substantial proportion of clinical MRSA strains *in vitro*, i.e., isolates which demonstrate enhanced susceptibility to standard β-lactam antibiotics (e.g., oxacillin) in the presence of NaHCO_3_. This phenotype correlates with increased MRSA clearance *in vivo* by standard β-lactam antibiotics, suggesting that patients with infections caused by such MRSA strains might be amenable to treatment with β-lactams. The mechanism(s) behind this phenotype is not fully understood but appears to involve *mecA*-PBP2a production and maturation axes. Our study adds significantly to this body of knowledge in terms of additional mechanistic targets of NaHCO_3_ in selected MRSA strains. This investigation demonstrates that NaHCO_3_ has direct impacts on S. aureus wall teichoic acid biosynthesis in NaHCO_3_-responsive MRSA. These findings provide an additional target for new agents being designed to synergistically kill MRSA using β-lactam antibiotics.

## INTRODUCTION

Methicillin-resistant Staphylococcus aureus (MRSA) strains have posed major treatment challenges since their first emergence in the 1960s (1, 2). As opposed to methicillin-susceptible S. aureus (MSSA), MRSA strains display “resistance” via standard *in vitro* testing conditions to most current β-lactam antibiotics (except for later-generation β-lactam agents, such as ceftaroline). Standard treatment guidelines recommend avoiding the use of early-generation β-lactams for MRSA infections, instead utilizing costlier, less effective, and/or more toxic treatment options (e.g., vancomycin, daptomycin, or linezolid) (3, 4). However, recent evidence suggests that a substantial proportion of MRSA strains may, in fact, be effectively treated by standard β-lactams, denoted by their intrinsic responsiveness to such agents in the presence of NaHCO_3_ (5–7).

This “NaHCO_3_-responsive” phenotype has been observed in a relatively large subset of clinical MRSA strains (~36% of strains in a collection of American bloodstream isolates) (8). Such strains display enhanced susceptibility to the early-generation β-lactams, cefazolin (CFZ) and oxacillin (OXA), in the presence of NaHCO_3_-supplemented media versus standard antimicrobial susceptibility testing (AST) media (5, 6, 8, 9). Strains exhibiting this phenotype appeared to produce less membrane-localized penicillin-binding protein 2a (PBP2a), the primary determinant of β-lactam resistance in MRSA. In addition, in the presence of NaHCO_3_, such responsive strains displayed a number of perturbations in genes involved in peptidoglycan (PG) synthesis and maturation (9, 10). These included (i) reduced membrane-localized PrsA (a chaperone required for proper PBP2a folding and functionality), (ii) reduced/altered expression of *pbp4*, *floA*, and carotenoids (components that make up the scaffolding upon which PBP2a matures and functions), and (iii) other genes involved in PG/cell wall synthesis (e.g., *ddh*, *pbp2*, and *sceD*) (9, 10). Further, certain genotypic variants of *mecA* (the gene encoding PBP2a) appeared to be associated with the NaHCO_3_-responsive phenotype via impacts on *mecA*/PBP2a expression and/or binding of PBP2a to β-lactams in the presence of NaHCO_3_ (11–13).

Wall teichoic acid (WTA) synthesis can have profound effects on MRSA susceptibility to β-lactam antibiotics (14–18). WTAs are polymers attached to the S. aureus cell wall PG that are involved in a variety of phenotypes, including virulence and pathogenesis, cell division, PBP localization, autolysis, and biofilm formation (15, 19–21). Synthesis of WTA is initiated by the enzyme TarO (also referred to as TagO) (22, 23) by catalyzing the transfer of *N*-acetylglucosamine-1-phosphate to a membrane-anchored undecaprenyl-phosphate carrier lipid (16, 21, 22). Further polymerization and glycosylation steps are carried out by the enzymes TarA, TarL, TarS, and TarM (among others) before the molecule is exported across the cell membrane by the translocator TarGH (19, 21). Once outside the cell, WTA is first linked to PG and then decorated with d-alanine via the actions of DltABCD to help regulate surface positive charge (19, 21, 24). Additionally, positively charged d-ala groups can be removed from lipoteichoic acid (LTA) by FmtA and transferred to WTA, thereby further modulating the cell surface charge and regulating various processes, including autolysis and cell division (25, 26).

Interference with WTA synthesis, either via inhibition with compounds like tunicamycin, ticlopidine, or targosil (14, 16, 17, 20) or through deletion of key WTA biosynthesis genes such as *tarO* and *fmtA*, has been shown to sensitize MRSA to selected β-lactams (16, 27). The principal mechanisms of β-lactam sensitization following WTA synthesis disruption are felt to be 3-fold. First, WTA and PG are linked via their shared requirement for undecaprenol for synthesis of both molecules (22). Interference with WTA synthesis may result in a buildup of undecaprenol-linked WTA intermediates, depleting undecaprenol precursors for use in the generation of PG; this disruption of PG biosynthesis potentially sensitizes cells to PG-inhibiting antibiotics, such as β-lactams (22). Second, glycosylated WTAs act as a scaffold for PG synthetic enzymes (15, 17, 22, 28); therefore, disruption of WTA synthesis could, in turn, also inhibit the function of crucial enzymes required to produce fully mature PG, also resulting in β-lactam sensitization. Third, d-ala-WTA inhibits AltA in the presence of protons (29, 30), thereby regulating autolytic activity. This has been linked to β-lactam activity (31).

**FIG 1:**
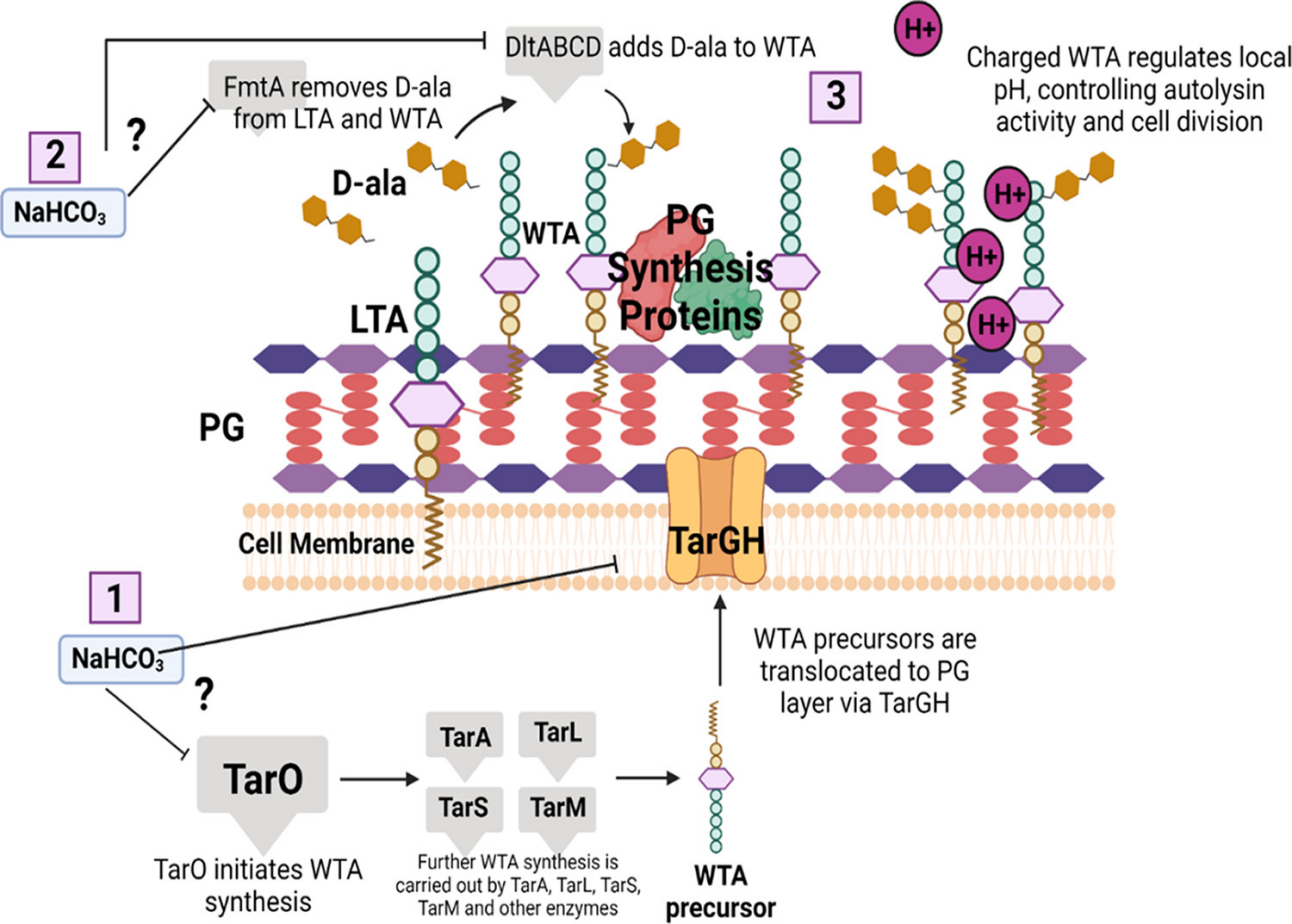
Model for potential points at which NaHCO_3_ may be impacting WTA synthesis, resulting in sensitization to β-lactams in responsive strains. (1) NaHCO_3_ may be inhibiting early WTA synthesis or WTA translocation to the outer cell layer. This could result in a buildup of WTA-linked undecaprenol precursors and a lack of scaffolding to support PG synthesis enzymes. (2) FmtA removes d-ala from LTA and makes d-ala available to WTA; inhibition of FmtA by NaHCO_3_ will reduce the amount of protonated d-ala on WTA, which is the physiological inhibitor of AtlA. Thus, NaHCO_3_ “inhibits the inhibitor,” ultimately enhancing autolytic activity. (3) A buildup of undecaprenol precursors, lack of PG enzyme scaffolding, and mislocalization of cell surface charge will lead to inhibition of PG synthesis and dysregulation of autolysis and cell division, ultimately resulting in β-lactam sensitization. This figure was created in BioRender.com.

The above-described intersecting pathways of PG and WTA synthesis and maturation are summarized in Fig. 1, as well as specific genes and biosynthetic steps where NaHCO_3_ may be impacting PG-WTA functionality to yield the β-lactam-NaHCO_3_-responsive phenotype. Overall, our findings support the notion that in NaHCO_3_-responsive strains, NaHCO_3_ inhibits the production of WTA, which is evidenced by reduced WTA content and size, enhanced autolysis, and aberrant cell division phenotypes in NaHCO_3_-responsive strains in the presence of NaHCO_3_. Inhibition of WTA production is known to sensitize MRSA to β-lactams and may be an integral part of the NaHCO_3_ responsiveness mechanism.

## RESULTS

### NaHCO_3_ synergy with PBP2-targeting β-lactams.

Previous work has demonstrated that inhibitors of early WTA synthesis, such as ticlopidine and tunicamycin, synergize with PBP2-targeting β-lactams in selected MRSA strains (16, 17, 20). Similarly, our prior studies demonstrated that NaHCO_3_ can sensitize selected MRSA strains to OXA and CFZ, β-lactams that more broadly target a range of PBPs, including PBPs 1, 2, and 3 (5, 8, 9, 32, 33). To assess whether NaHCO_3_ (like ticlopidine) synergizes with the specific PBP2-targeting β-lactam, cefuroxime (16, 34), the MICs of cefuroxime were determined for NaHCO_3_-responsive and -nonresponsive MRSA strains in the presence and absence of 44 mM NaHCO_3_. This concentration of NaHCO_3_ has been demonstrated to be the optimal concentration for disclosing β-lactam-susceptible phenotypes in a previous dose-response study (5). As expected, when exposed to cefuroxime in the absence of NaHCO_3_, all four strains were highly resistant (Table 1). However, when exposed to cefuroxime in the presence of NaHCO_3_, only the two previously defined NaHCO_3_-responsive strains (MRSA 11/11 and MW2) were sensitized to cefuroxime, with 64-fold and 16-fold decreases in MICs, respectively; the two NaHCO_3_-nonresponsive strains (COL and BMC1001) remained highly resistant to cefuroxime under these conditions (Table 1).

**TABLE 1:** MICs of cefuroxime in CA-MHB with and without NaHCO_3_ and ticlopidine

Strain	Cefuroxime MICs (μg/mL)			Cefuroxime with 32 μg/mL ticlopidine MICs (μg/mL)		
	CA-MHB	CA-MHB-Tris	CA-MHB-Tris with 44 mM NaHCO_3_	CA-MHB	CA-MHB-Tris	CA-MHB-Tris with 44 mM NaHCO_3_
MRSA 11/11	32	256	4	8	8	8
MW2	16	128	8	8	8	8
COL	>512	>512	512	>512	>512	>512
BMC1001	>512	>512	>512	>512	>512	>512

As NaHCO_3_ appeared to selectively sensitize MRSA strains to cefuroxime, we next assessed whether the known early WTA synthesis inhibitor, ticlopidine (16), exerted broad or selective synergy with cefuroxime in the four prototype MRSA strains. Interestingly, MIC testing revealed that ticlopidine, as seen with NaHCO_3_ alone, only sensitized NaHCO_3_-responsive (but not NaHCO_3_-nonresponsive) MRSA strains to cefuroxime (Table 1). Of note, combined exposures to ticlopidine and NaHCO_3_ did not further enhance sensitization to cefuroxime (Table 1), suggesting that both agents may be targeting the *tarO*-mediated early WTA biosynthetic step.

### Impacts of NaHCO_3_ on WTA expression.

**FIG 2:**
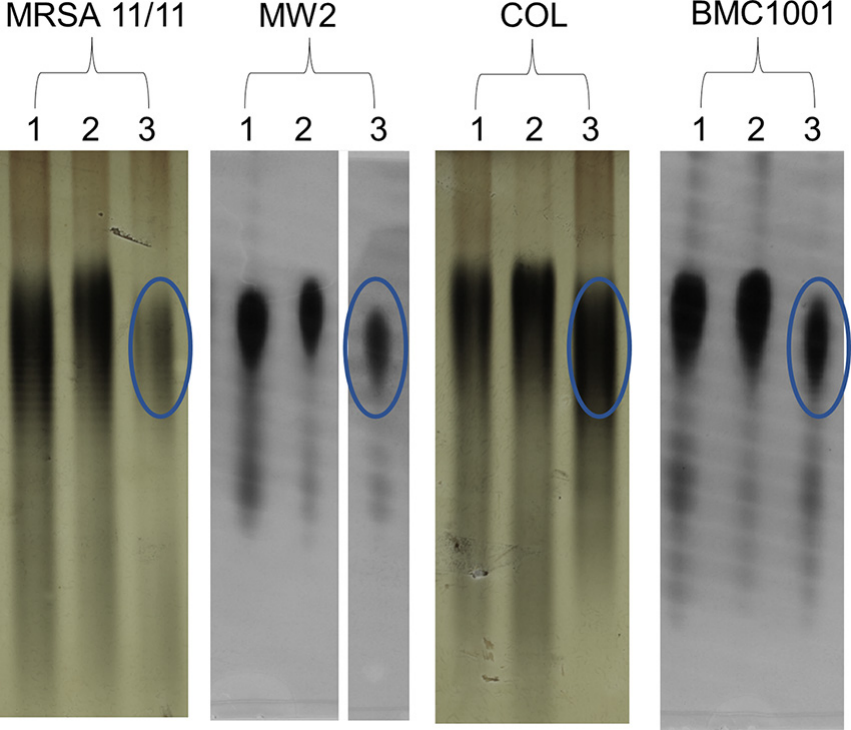
WTA profile of S. aureus MRSA11/11, MW2, COL, and BM1001 analyzed by SDS-PAGE. 1, CA-MHB; 2, CA-MHB plus OXA; 3, CA-MHB plus 44 mM NaHCO_3_. OXA exposures are equivalent to 1/2× the MIC under the indicated condition. These studies were designed to compare the impacts of OXA alone versus NaHCO_3_ alone on WTA production.

To assess the direct impact of NaHCO_3_ on WTA expression, extracts of total WTA from the two NaHCO_3_-responsive and the two NaHCO_3_-nonresponsive MRSA strains grown in the presence and absence of NaHCO_3_ were compared by gel electrophoresis. Cells grown in plain media or media supplemented with 1/2× MIC of OXA displayed large quantities of WTA of similar molecular size; WTA from the NaHCO_3_-responsive strains (11/11 and MW2), when exposed to OXA, appears to have a slightly higher molecular weight than when grown in plain medium, a behavior not observed for the NaHCO_3_-nonresponsive strains (Fig. 2). Interestingly, when exposed to NaHCO_3_, all strains produced WTAs of a slightly smaller molecular weight; however, only the NaHCO_3_-responsive strains MRSA 11/11 and MW2 showed an obvious decrease in both molecular weight and quantity of WTA being produced (Fig. 2), with the effect being most striking in the MRSA 11/11 strain.

### Effects of NaHCO_3_ on cell division and autolysis.

The WTA synthesis pathway begins with the TarO enzyme, which catalyzes the formation of C55-PP-GlcNAc from P-GlcNAc (14, 16, 21, 22). Deletion of *tarO* results in cells that are deficient in WTA and are defective in several cell division-associated phenotypes (e.g., division-septation), as well as in autolysis (16, 17, 19, 20). Given the data described above, suggesting that NaHCO_3_ can target the *tarO*-mediated WTA biosynthetic step, our MRSA strains were assessed for septal division patterns by transmission electron microscopy (TEM), as well as extents of Triton-X-induced autolysis following exposure to NaHCO_3_.

**FIG 3:**
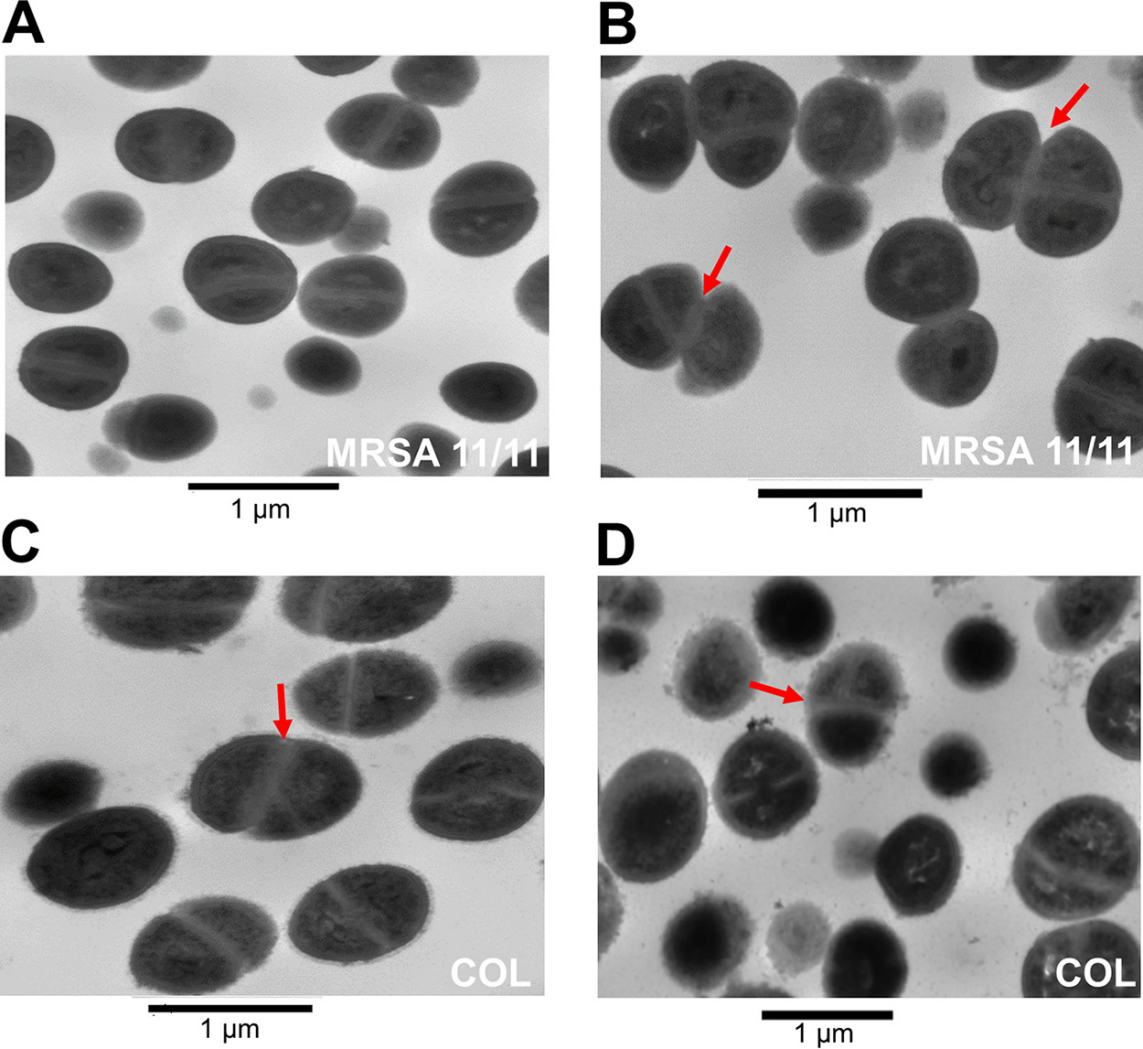
Impact of NaHCO_3_ on cell division in NaHCO_3_-responsive and -nonresponsive strains. Representative images of division septa at 8 h in the presence of OXA (MRSA 11/11) (A), NaHCO_3_ and OXA (MRSA 11/11), OXA (COL) (C), and NaHCO_3_ and OXA (COL) (D). OXA exposures are equivalent to 1/2× the MIC under the indicated condition. Red arrows point to cell clusters with septa at nonorthogonal division planes.

Previously, TEM analysis revealed that Δ*tarO* deletion MRSA strains display aberrant division septa, where new septa form at nonorthogonal angles to previously formed septa, and newly divided cells fail to properly separate, resulting in multicellular clusters (15, 17). TEM was performed on a NaHCO_3_-responsive strain, MRSA 11/11, and nonresponsive strain, COL, grown in the presence of 1/2× MIC of OXA, with or without NaHCO_3_. In the presence of OXA alone, no aberrant septal patterns were observed in MRSA 11/11 (Fig. 3A). However, in the presence of NaHCO_3_ and OXA, MRSA 11/11 cells began to display septal formation at nonorthogonal angles, and multicellular clustering was observed in ~18% of cells within selected visual fields (out of 71 cells observed undergoing active division) (Fig. 3B); this is similar to patterns previously observed in Δ*tarO* strains (15, 17). In contrast, fewer COL cells exposed to OXA alone or in combination with NaHCO_3_ displayed such aberrant septal patterns and multicellular clustering (~12% and 5%, respectively, out of ~60 cells observed undergoing active division under each condition) (Fig. 3C and D). Also, a significantly higher number of cell clusters in active separation were observed in MRSA 11/11 than in COL in the presence of NaHCO_3_ and OXA (*, *P* = 0.01).

**FIG 4:**
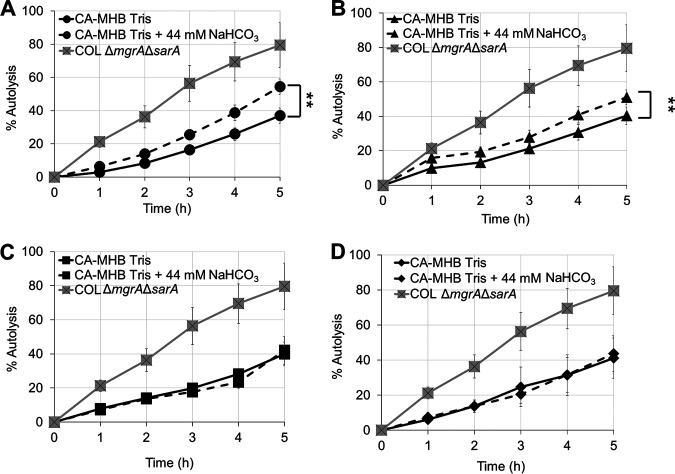
Impact of NaHCO_3_ on Triton-X-induced autolysis in NaHCO_3_-responsive strains MRSA 11/11 (A) and MW2 (B) and nonresponsive strains COL (C) and BMC1001 (D). Cells were grown in CA-MHB-Tris with or without 44 mM NaHCO_3_ prior to exposure to Triton-X. COL Δ*mgrA* Δ*sarA* was used as a control. Statistics calculated on area under the curve (AUC) by Student’s *t* test; **, *P* < 0.01. Data are the result of six independent replicates for each strain or condition.

WTAs play a critical role in regulating the activity of autolysins, likely by governing the local pH at the cell surface with the formation of proton-dense regions, which can interfere with autolytic function (30, 35). In the absence of WTA, these proton-dense pockets are missing, resulting in higher activity of the major autolysins and increased rates of Triton-X-induced autolysis (15, 17, 20, 30). To assess the impact of NaHCO_3_ on autolysis, NaHCO_3_-responsive and -nonresponsive MRSA strains were grown in the presence or absence of NaHCO_3_ and then exposed to Triton-X and quantified for the extent of autolysis over time. Following exposure to NaHCO_3_, responsive MRSA strains demonstrated significantly increased extents of autolysis (as assessed by area under the curve [AUC] calculations) versus cells that were not preexposed to NaHCO_3_ (Fig. 4A and B). This observation is in accordance with the higher rate of daughter cell separation previously observed by TEM analysis. The presence of WTA is known to exclude the presence of the major autolysin, Atl, targeting this peptidoglycan hydrolase exclusively to the cross-wall region (35). Thus, a lower content of WTA (as observed for MRSA 11/11 in the presence of NaHCO_3_) may explain the higher rate of cell separation than COL. Comparatively, NaHCO_3_ exposure had no impact on autolysis profiles in nonresponsive MRSA strains (Fig. 4C and D).

Both WTA and PG can contribute to alterations in cell division and autolysis (17, 36–38), and their assembly pathways are known to be interconnected. We thus determined the impacts of NaHCO_3_ on PG composition in our NaHCO_3_-responsive and -nonresponsive strains; this allowed assessment of whether the observed cell division and autolysis phenotypes described above were more likely due to the NaHCO_3_ impact observed on WTA or if alterations also occurred on PG. High-performance liquid chromatography (HPLC) analysis revealed that exposure to NaHCO_3_ stimulated a modest increase in *O*-acetylation, while exposure to OXA resulted in approximately twice the *O*-acetylation level, as measured by release of acetic acid, in all four strains (see Fig. S1 and Table S1 in the supplemental material). In the presence of OXA and NaHCO_3_, no consistent patterns of alterations to *O*-acetylation were observed among the four strains compared to *O*-acetylation levels obtained in the presence of OXA (Fig. S1; Table S1). HPLC analysis of PG muropeptide species revealed identical profiles for all four strains in standard media (Fig. S2). While exposure to NaHCO_3_ did not result in any alteration to the elution profile, exposure to OXA or NaHCO_3_ in combination with OXA resulted in a similar reduction in PG cross-linking for all four strains (Fig. S2). Collectively, these data indicated that differential NaHCO_3_-stimulated impacts on cell division are unlikely to be due to changes in PG composition but, rather, a WTA-specific effect.

### Role of NaHCO_3_ in modulating transcription and translation of genes involved in early WTA synthesis, translocation, and maturation.

**FIG 5:**
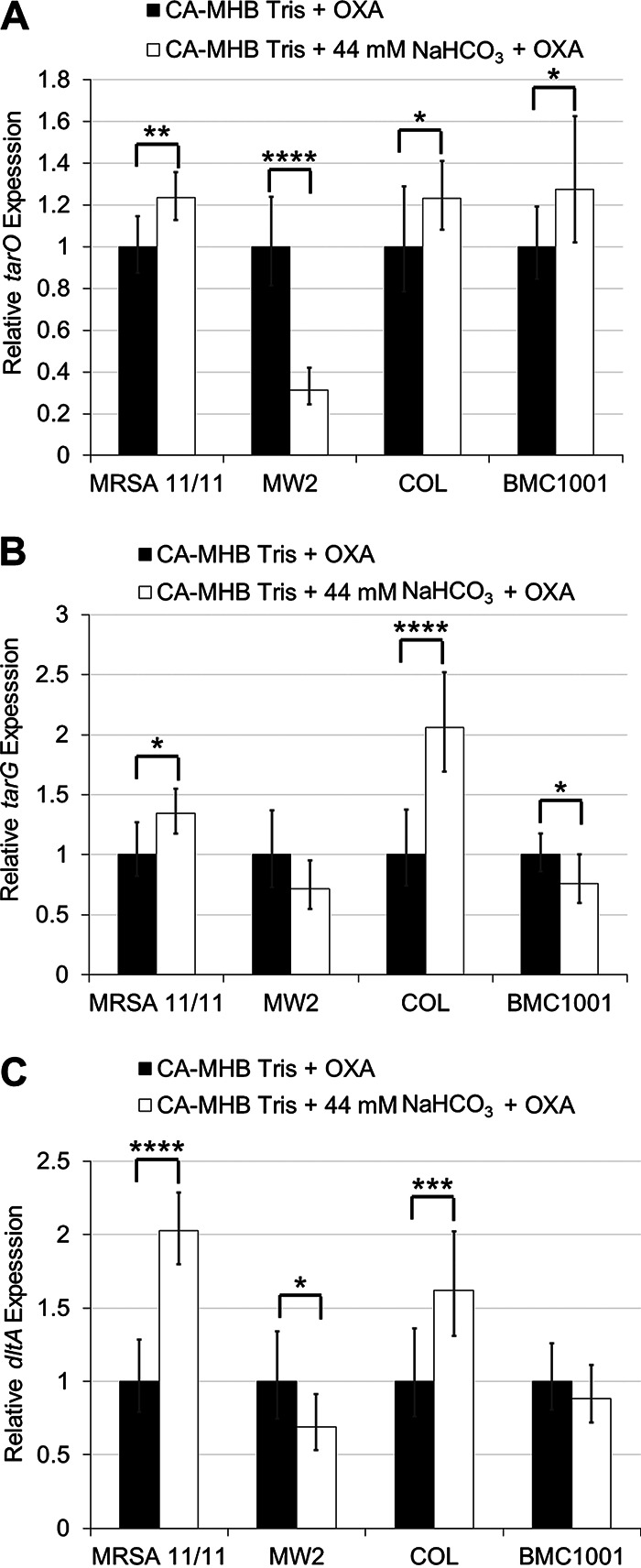
Expression of WTA synthesis, translocation, and modification genes in the presence and absence of NaHCO_3_ in NaHCO_3_-responsive and -nonresponsive strains. (A) *tarO* (early WTA synthesis); (B) *tarG* (WTA translocation); (C) *dltA* (WTA modification). OXA exposures are equivalent to 1/2× the MIC under the indicated condition. Statistics calculated by a Student's *t* test; *, *P* < 0.05; **, *P* < 0.01; ***, *P* < 0.001; ****, *P* < 0.0001. Data are the result of two biological replicates performed in technical triplicates in at least two independent assays for each strain or condition.

**FIG 6:**
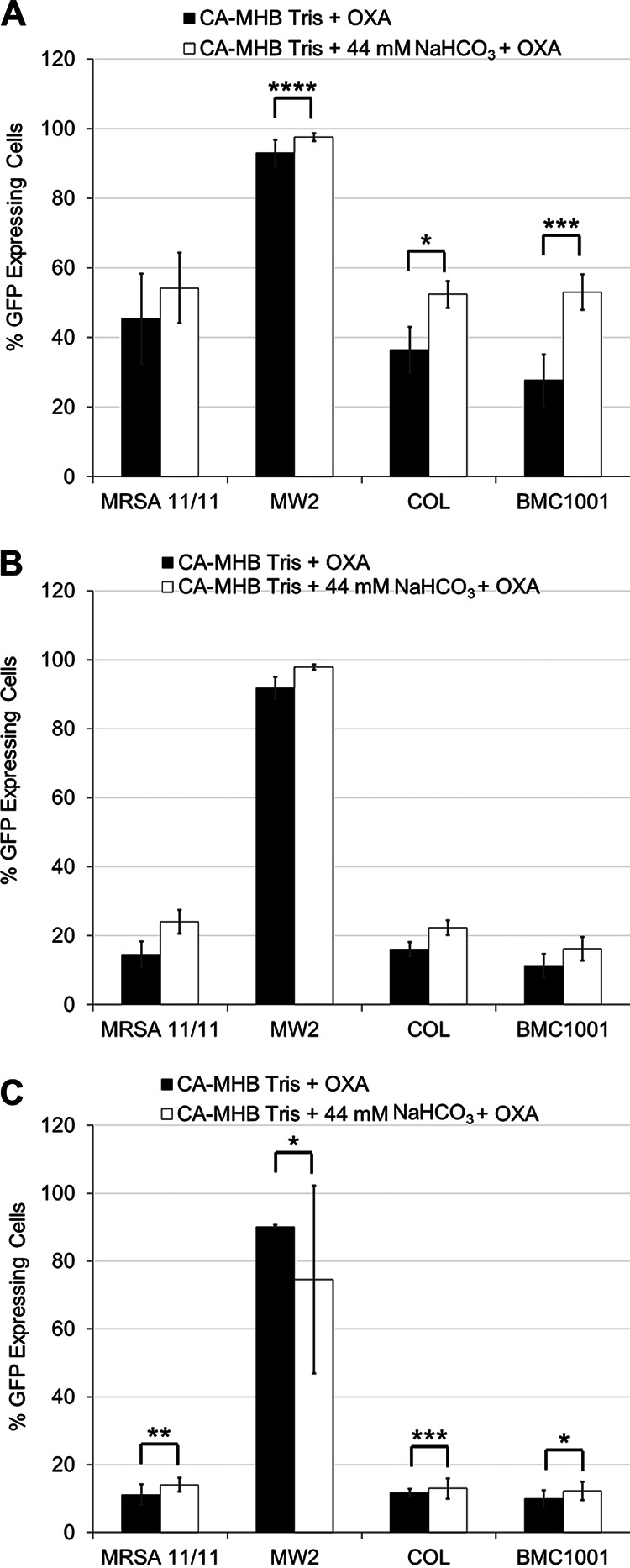
Translation of WTA synthesis, translocation, and modification genes. Flow cytometry was used to detect GFP expression in *tarO* (A), *tarG* (B), and *dltA* (C) translational fusions grown in the presence and absence of NaHCO_3_, as measured by the percentage of cells expressing GFP in a population of 10,000 cells. Flow readings were taken after 3 h of incubation under the indicated condition. OXA exposures are equivalent to 1/2× the MIC under the indicated condition. Statistics calculated by Student’s *t* test; *, *P* < 0.05; **, *P* < 0.01; ***, *P* < 0.001. Data are the result of six independent replicates for each strain or condition.

The effect of NaHCO_3_ on transcription of *tarO*, *tarG*, and *dltA* (23) was assessed by reverse transcription-quantitative PCR (qRT-PCR). Interestingly, NaHCO_3_ did not selectively repress expression of any of the genes tested in NaHCO_3_-responsive versus -nonresponsive strains in the presence of OXA (Fig. 5A to C). Next, green fluorescent protein (GFP) fusions for the same three genes were assessed via flow cytometry for translational profiles following growth in media with or without NaHCO_3_. As was observed with the transcriptional data, NaHCO_3_ did not significantly and selectively repress translation of any gene at either the 3-h or 6-h growth time point in any strain tested (Fig. 6A to C; Fig. S3A to C). Similar results were obtained for cells grown in the absence of OXA (data not shown).

**FIG 7:**
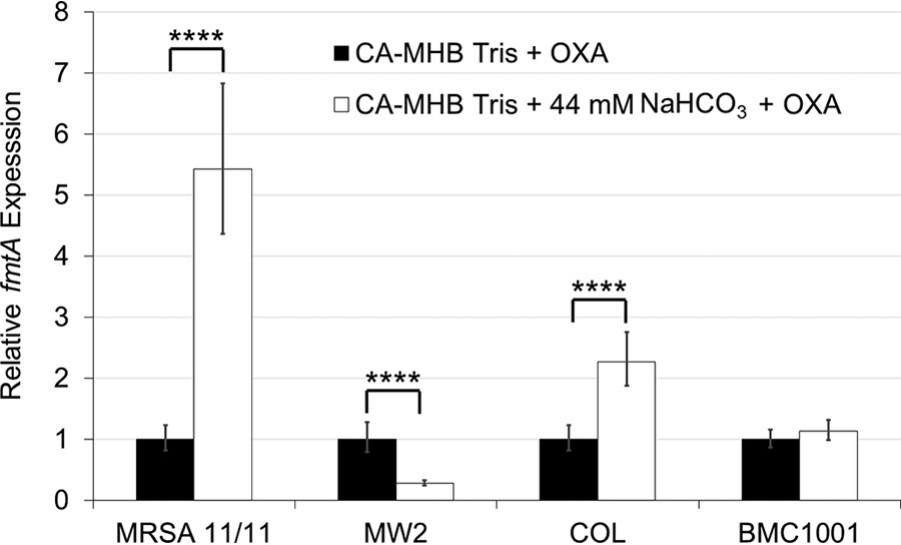
Expression of WTA equilibrium gene, *fmtA*, in the presence and absence of NaHCO_3_ in NaHCO_3_-responsive and -nonresponsive strains. OXA exposures are equivalent to 1/2× the MIC under the indicated condition. Statistics calculated by Student’s *t* test; ****, *P* < 0.0001. Data are the result of two biological replicates performed in technical triplicates in at least two independent assays for each strain or condition.

In addition to the impacts of NaHCO_3_ on genes known to be involved directly in WTA synthesis and modification, we also assessed the impact of NaHCO_3_ on expression of *fmtA*, a gene involved in regulating WTA equilibrium by acting as a carboxypeptidase in the transfer of d-ala groups between WTA and LTA molecules (25, 26). Inactivation of *fmtA* has also been demonstrated to enhance β-lactam susceptibility and autolysis rates (26, 27). Analysis of *fmtA* transcription in the presence and absence of NaHCO_3_ revealed that among responsive strains, *fmtA* expression was substantially and significantly enhanced in MRSA 11/11 while being repressed in MW2 (Fig. 7). NaHCO_3_ had no impact on expression of *fmtA* in nonresponsive strain BMC1001 and significantly enhanced expression of *fmtA* in nonresponsive strain COL (Fig. 7).

## DISCUSSION

The ability of NaHCO_3_ to sensitize MRSA to cell wall/cell membrane-active antimicrobials is now a well-established phenomenon (5, 6, 39). Of note, previous work by Dorschner et al. found that the carbonate ion was the key factor in tissue culture media capable of sensitizing MRSA to the membrane-targeting antimicrobial peptide LL-37 (39). This effect was found to be independent of the specific carbonate salt, as well as of the medium pH (39). As the mechanism of action of positively charged LL-37 is via membrane disruption (40), it is possible that one pathway by which NaHCO_3_ may sensitize cells to LL-37 is via alterations to surface charge and/or cell wall content, allowing better access of LL-37 to the cell membrane. Therefore, it is likely that the one consensus mechanism of sensitization to both membrane-disrupting agents such as LL-37 and PG synthesis-disrupting β-lactams occurs via impacts of NaHCO_3_ on specific steps in PG and WTA synthesis.

NaHCO_3_ has previously been demonstrated to impact multiple aspects of PG synthesis (5, 9, 12), which may contribute to the overall β-lactam-sensitizing effect exhibited by selected MRSA strains in the presence of NaHCO_3_. PG and WTA syntheses are intimately coupled, and disruptions in WTA synthesis can lead to dysfunction in PG synthesis and, ultimately, β-lactam sensitization (15–17, 22). In the present study, we investigated the impact of NaHCO_3_ on WTA synthesis as a potential contributing mechanism to the NaHCO_3_ responsiveness phenotype.

Several interesting observations emerged from these studies. We demonstrated that NaHCO_3_ selectively sensitized NaHCO_3_-responsive MRSA to cefuroxime, a PBP2-targeting β-lactam known to synergize with specific WTA synthesis inhibitors (16). These data suggested that the ability of NaHCO_3_ to impact WTA synthesis involved specific genetic targets in responsive strains rather than global biochemical impacts on this pathway. Further, we demonstrated that strains displaying selective sensitization to OXA in the presence of NaHCO_3_ were also selectively sensitized to cefuroxime when combined with NaHCO_3_. This finding likely points to a common underlying mechanism of NaHCO_3_ sensitization for both of these β-lactams. Indeed, a recent screen of 28 NaHCO_3_-responsive and -nonresponsive MRSA isolates revealed that 21 out of 28 strains displayed concordant cefuroxime-OXA-responsive and -nonresponsive phenotypes, with 13/16 OXA-responsive strains also being responsive to cefuroxime (S. C. Ersoy and A. S. Bayer, unpublished data; not shown). Moreover, in the present study, the TarO inhibitor ticlopidine also selectively sensitized NaHCO_3_-responsive MRSA to cefuroxime, indicating that NaHCO_3_ may have a similar target and/or mechanism of action as ticlopidine. The deletion of the *tarO* gene is described to have an impact on the cooperative function of PBP2 and PBP4 (16), while WTAs are suggested to have an important role in localizing PBP4. In fact, our recent data sets demonstrated a selective repression in *pbp4* expression in NaHCO_3_-responsive versus -nonresponsive strains (9). However, we did not observe any alterations in the cross-linking level of the PG when comparing NaHCO_3_-responsive versus -nonresponsive strains in the presence of NaHCO_3_, as would be expected from the impairment of PBP4 activity. In this way, it is possible that the impact of NaHCO_3_ on WTA synthesis occurs at a later stage. This hypothesis does not exclude the possibility that NaHCO_3_ also targets TarO and that the PG cross-linking is maintained by a compensatory mechanism. Of note, in the presence of NaHCO_3_, the quantity of WTA produced is lower only for the responsive strains, while the molecular size of WTA is lower for both responsive and nonresponsive strains, suggesting that NaHCO_3_ may target multiple steps of WTA biosynthesis.

As mentioned above, direct measurement of the impact of NaHCO_3_ on WTA production demonstrated notable effects on both the molecular size and apparent quantity of WTA produced by NaHCO_3_-responsive MRSA strains. Interestingly, the most pronounced outcomes of NaHCO_3_ on WTA content appeared in the CC8/ST8/USA300 strain background (MRSA 11/11). Previous work has demonstrated that strains of this genetic background are more likely than other common genotype strains to display a NaHCO_3_-responsive phenotype and tend to show higher degrees of sensitization to β-lactams than other clonotypes (5, 8, 11). This correlation implies that strains of the CC8 genotype may be more susceptible to the impacts of NaHCO_3_ on WTA synthesis, resulting in increased rates of NaHCO_3_ responsiveness and higher degrees of β-lactam sensitization.

Disruption of WTA synthesis can have profound impacts on cell division by altering the scaffolding necessary for the function of PG synthetic enzymes, as well as perturbing the surface charge profiles required to regulate the activity of autolysins. Thus, cells that are disrupted in WTA synthesis, either through mutation of key WTA synthesis genes or exposure to WTA synthesis-inhibiting compounds, tend to display aberrant division septa and increased rates of autolysis (17, 20). In this study, we observed that NaHCO_3_-responsive strain MRSA 11/11 demonstrated both these phenotypes in the presence of NaHCO_3_, two hallmarks of WTA synthesis disruption. In contrast, TEM analysis of the nonresponsive strain, COL, revealed a notable decrease in such aberrant division septa in the presence versus absence of NaHCO_3_ and no enhancement of autolysis. We recognize that inhibition of the PG biosynthesis enzymes PBP1 and PBP4 usually results in changes to the PG cross-linking level (36, 38) and that these alterations can, in turn, impact cell division and autolysis. However, in the current study, NaHCO_3_ did not appear to have any differential impacts on two major metrics of PG composition (*O*-acetylation and muropeptide species profiles) between NaHCO_3_-responsive and -nonresponsive strains. These data suggested that the observed and selective impacts of NaHCO_3_ on cell division by TEM and autolysis in NaHCO_3_-responsive strains can plausibly be attributed to differential and selective impacts of NaHCO_3_ on WTA content and/or structure, rather than alterations to PG composition. Verification of these outcomes will require additional MRSA strains to be assessed similarly.

We quantified the impacts of NaHCO_3_ on both transcriptional and translational profiles of several key genes involved in WTA synthesis, modification, and equilibrium (*tarO*, *tarG*, *dltA*, and *fmtA*). These studies revealed that exposure to NaHCO_3_ did not selectively repress expression or translation of any of these genes in NaHCO_3_-responsive versus -nonresponsive MRSA. This raises two possibilities, (i) NaHCO_3_ may be acting to repress transcription and/or translation of other gene(s) involved in the regulation of WTA synthesis, or (ii) NaHCO_3_ is impacting one of the genes assessed here at the posttranslational or enzymatic functional level. Our recent RNA sequencing comparisons of NaHCO_3_ exposures in NaHCO_3_-responsive versus NaHCO_3_-nonresponsive MRSA were concordant with the above transcriptional data by qRT-PCR (10). None of the WTA biosynthetic genes that were evaluated in this current investigation were differentially expressed in the presence of NaHCO_3_ exposures in these two phenotype groups. Interestingly, the mechanism of ticlopidine, a compound which appears to closely mimic the impacts of NaHCO_3_ on the early *tarO*-dependent step in WTA biosynthesis, is posttranscriptional/translational at the protein functionality level; this involves directly inhibiting the enzymatic activity of TarO in catalyzing the conversion of GlcNAc and undecaprenyl-P into undecaprenyl-P-P-GlcNAc (16). Further work is needed to understand what precise posttranslational effects that NaHCO_3_ may be exerting are in play in terms of impacting the functionality of specific WTA synthetic enzymes.

Previously, NaHCO_3_ was shown to selectively reduce the amount of membrane-localized PBP2a and PrsA in NaHCO_3_-responsive strains (9), as well as selectively reduce expression of *pbp4*, which is required for highly cross-linked PG in certain MRSA strains (41–43). Further, NaHCO_3_ was capable of altering expression of components required for the formation of functional membrane microdomains (FMMs) (9), an integral part of the scaffolding upon which PG synthetic proteins function (44). In addition to FMMs, WTAs provide additional scaffolding to stabilize PG synthetic proteins, including PBP4 (19, 22, 28). Our current data suggest a more refined spatial model of potential NaHCO_3_ impacts on PG synthetic machinery, whereby NaHCO_3_ disrupts WTA production in NaHCO_3_-responsive strains, reducing the formation of the scaffolding required for PG synthesis. The proposed cumulative outcomes of selective WTA disruption in NaHCO_3_-responsive MRSA would be a reduction in membrane-localized PBPs, aberrant cell division, increased autolysis, and enhanced susceptibility to multiple β-lactams (e.g., CFZ, OXA, and cefuroxime). The precise mechanism(s) by which NaHCO_3_ is interfering with WTA production and how specific genetic backgrounds influence this phenotype are currently under study.

Our investigation had several limitations. As noted above, our conclusions are based on study of only four prototype MRSA strains and need to be verified in larger strain sets of both NaHCO_3_-responsive and -nonresponsive strains. Also, we only examined the selective impacts of NaHCO_3_ on two metrics of PG functionality; a more detailed analysis of NaHCO_3_’s effects on other PG biosynthetic parameters are required. Moreover, total WTA was only semiquantified by gel assay in comparing the NaHCO_3_-responsive versus -nonresponsive strains; a more systematic, quantitative, and structural assessment of the WTA components will be necessary (e.g., by mass spectrometry-nuclear magnetic resonance (MS-NMR] techniques [45]). Further, an investigation into the effects of NaHCO_3_ on the function of WTA biosynthetic enzymes selectively impacted in NaHCO_3_-responsive strains (e.g., TarO) would be important. Finally, a more in-depth investigation into the effect of NaHCO_3_ on other genes involved in cell wall division (e.g., *ftsZ*, *pbp1*, and *ezrA* [36, 46, 47]) is also warranted.

Overall, this study provides the first evidence for implicating disruptions of WTA synthesis as a contributing mechanism of NaHCO_3_ sensitization to β-lactams; i.e., via reductions in WTA content, enhanced autolysis, and alterations in cell division dynamics. In combination with previous data sets, a potential model is emerging where NaHCO_3_ inhibits WTA synthesis, resulting in reduced localization/functionality of essential PG biosynthetic enzymes, ultimately sensitizing NaHCO_3_-responsive MRSA strains to β-lactams.

## MATERIALS AND METHODS

### Bacterial strains and growth conditions.

The following strains used in the study are four well-characterized prototype MRSA strains derived from patients with clinical infections: MRSA 11/11 (USA300), MW2 (USA400), COL (USA100), and BMC1001 (USA500), previously classified as either NaHCO_3_ responsive (MRSA 11/11 and MW2) or nonresponsive (COL and BMC1001) based on prior *in vitro* MIC testing with the standard β-lactams, CFZ and OXA (5). Strains were stored at −80°C until they were thawed for use and isolated on tryptic soy agar (TSA). For all assays, unless otherwise indicated, strains were grown in cation-adjusted Mueller-Hinton Broth (CA-MHB; Difco) with or without 100 mM Tris buffer (pH 7.3 ± 0.1) and with or without 44 mM NaHCO_3_. The Tris buffer was added to maintain a stable pH in ambient air upon addition of NaHCO_3_. To minimize buffering between HCO_3_^−^ and CO_2_ present in ambient air, NaHCO_3_ was added fresh to an aliquot of CA-MHB-Tris on the day of the experiment, and the pH was readjusted with HCl. This 44 mM NaHCO_3_ is the concentration present in the tissue culture medium DMEM and, importantly, has been found to be the optimal concentration for disclosing the NaHCO_3_-responsive phenotype to CFZ and OXA *in vitro* (5). Further, this concentration of HCO_3_^−^ is reflective of those observed in visceral tissues (48). For assays in which cells were exposed to OXA, a concentration equivalent to 1/2× the MIC of OXA under the indicated condition was used (Table S2). Also, 2% NaCl was added to all media containing OXA.

### Determination of MICs.

MICs were determined as previously described (5). Briefly, cells were grown overnight in the indicated testing condition and then diluted to 5 × 10^6^ CFU/mL with 2-fold serial dilutions of cefuroxime (Sigma-Aldrich) in the same medium and incubated overnight without aeration at 37°C. For ticlopidine synergy assays, a final concentration of 32 μg/mL ticlopidine (Sigma-Aldrich) was included in media with 2-fold serial dilutions of cefuroxime. This ticlopidine concentration was established as optimum for these MIC assays based on extensive pilot testing. The MIC was scored as the first well in which visual turbidity was decreased compared to the no-drug control well.

### WTA isolation and gel electrophoresis.

Cells were grown overnight in CA-MHB with and without NaHCO_3_. These cultures were used to inoculate CA-MHB with and without NaHCO_3_ and CA-MHB plus 2% NaCl with 1/2× MIC of OXA to an initial optical density at 620 nm (OD_620_) of 0.0006. These three exposure groups were designed to look at the impact of NaHCO_3_ exposure alone (in the absence of OXA) on WTA production. The cells were harvested by centrifugation to the final OD_620_ of 1, washed once with 30 mL of buffer 1 (50 mM 2-*N*-morpholinoethanesulfonic acid [MES], pH 6.5), and suspended in 30 mL of buffer 2 (4% [wt/vol] sodium dodecyl sulfate [SDS], and 50 mM MES, pH 6.5). Samples were placed in a boiling water bath for 1 h, and the cells were collected by centrifugation (7,000 × *g*, 15 min). The pellet was suspended in 2 mL of buffer 2, transferred to a microcentrifuge tube, and centrifuged (13,000 × *g*, 10 min). Next, the pellet was washed once with 2 mL of buffer 2, once with 2 mL of buffer 3 (2% NaCl and 50 mM MES, pH 6.5), and finally with 2 mL of buffer 1. After the last wash, samples were treated with proteinase K solution (20 mM Tris-HCl [pH 8.0], 0.5% [wt/vol] SDS, and 20 μg/mL of proteinase K) and incubated at 50°C for 4 h with agitation. Following digestion, samples were washed once with buffer 3 and at least three times with distilled H_2_O to remove the SDS. Samples were thoroughly resuspended in 0.1 M NaOH and incubated at 25°C with agitation for 16 h to hydrolyze the WTA. Insoluble cell wall debris was removed by centrifugation (13,000 × *g*, 10 min), and the supernatant containing the hydrolyzed WTA was directly analyzed by polyacrylamide gel electrophoresis (PAGE).

To visualize the WTA by gel electrophoresis, the samples were neutralized with 0.15 M Tris base, pH 7.8, and were analyzed in an acrylamide gel (separating gel, 30% total acrylamide [T], 6% bisacrylamide [C]; stacking gel, 3% T, 0.26% C) using a Bio-Rad Protean II xi cell. Gels were run at 4°C for 21 h at a constant current (20 mA with two gels) in Tris-Tricine running buffer (0.1 M Tris base and 0.1 M Tricine, pH 8.2). WTA bands were visualized using the alcian blue-silver staining procedure.

### TEM.

To prepare cells for TEM, MRSA 11/11 and COL were grown overnight in 1 mL of CA-MHB-Tris or CA-MHB-Tris plus NaHCO_3_ at 37°C with aeration. Fifty microliters of each overnight culture was diluted into 1 mL of the same overnight growth medium (CA-MHB-Tris or CA-MHB-Tris plus NaHCO_3_) containing 2% NaCl and 1/2× MIC of OXA and incubated for 8 h at 37°C with aeration. Cells were washed twice with phosphate-buffered saline (PBS) and pelleted. We added 500 μL of 2.5% glutaraldehyde on top of each pellet to fix samples prior to TEM. Cells were imaged at a magnification of ×8,000 (model 100CX; Jeol, Tokyo, Japan) using digital image capture.

### Triton X-100-induced autolysis.

Cells were grown overnight in either CA-MHB-Tris or CA-MHB-Tris plus NaHCO_3_ and then diluted into 30 mL of the same medium and grown at 37°C with aeration to an OD_580_ of 0.5. Cells were washed twice in PBS and then resuspended in 50 mM Tris-HCl buffer, pH 7.2, to a final OD_580_ of 0.7. For determination of the extent of autolysis, 10 mL of washed and diluted cells were mixed with 5 μL of Triton X-100 and incubated at 30°C with aeration. Cell lysis was determined by measuring the OD_580_ at 0-, 1-, 2-, 3-, 4-, and 5-h time points. The area under the curve (AUC) was calculated by linear approximation, and the AUCs for individual curves were compared by Student’s *t* test.

### RNA isolation and qRT-PCR.

RNA was isolated as previously described (5). Briefly, cells were grown to log phase (OD_600_ = 0.5) in the indicated medium. Cells were pelleted and disrupted by mechanical disruption (FastPrep Lysing Matrix B; MP Bio) with 1% β-mercaptoethanol. RNA was isolated with the RNeasy kit (Qiagen), then DNA was removed with Turbo DNase (Invitrogen), and final RNA was concentrated with the RNA cleanup and concentrator kit (Genesee). RNA was reversed transcribed to cDNA with SuperScript reverse transcriptase (Invitrogen). For qRT-PCR, *gyrB* was used as a housekeeping gene to normalize transcript quantifications, and relative quantification was normalized using the threshold cycle (ΔΔ*C_T_*) method. Sequences for *tarO*, *tarG*, *dltA*, *fmtA*, and *gyrB* primers can be found in Table S3 in the supplemental material. All qRT-PCR gene expression data were determined from two biological replicates per strain per condition, performed in technical triplicates. For each strain, gene expression was normalized to expression obtained in CA-MHB-Tris, with this value set equal to 1.

### Construction of *tarO*, *tarG*, and *dltA* translational fusions and flow cytometry.

To determine translational activities of the *tarO*, *tarG*, and *dltA* genes using a reporter gene, *gfp*, the upstream region, including the promoter, was cloned from the respective genes before the translational start code (ATG) of the *gfp* reporter gene in a shuttle plasmid pALC1484 (49). First, the pALC1484 vector was modified by removing the ribosome-binding site (RBS), along with the spacing region between the RBS and start codon ATG of the *gfp* gene and replaced with the RBS and spacing of the respective genes (e.g., *tarO*, *tarG*, and *dltA*) using pairwise primers (Fig. S4; Table S4) flanking with EcoRI and XbaI sites and template DNA as pALC1484 by PCR. The 194-bp, 204-bp, and 221-bp promoter fragments flanking with EcoRI and XbaI sites of the *tarO*, *tarG*, and *dltA* genes without the RBS, respectively, were PCR amplified and cloned into the respective modified pALC1484 vector fragments in the Escherichia coli IM08B strain (50). Final constructs were verified by enzymatic digestion and DNA sequencing and mobilized into various strains (e.g., MW2, MBC1001, MRSA11/11, and COL [Table S5]) by electroporation and selected on TSA with chloramphenicol (10 μg/mL).

For flow cytometry, strains were grown overnight in the indicated medium (CA-MHB-Tris ± 44 mM NaHCO_3_) at 37°C with aeration and then diluted 1:10 into the same medium with or without 2% NaCl and 1/2× MIC of OXA. Cells were incubated at 37°C with aeration, and aliquots were taken at 3 h and 6 h to be assessed by flow cytometry. At the indicated time points, 100 μL of cells was diluted into 2 mL of PBS, and 10,000 cells were then analyzed by flow cytometry on FACSCalibur (Becton Dickinson). The percentage of cells expressing GFP in each sample was analyzed with FlowJo software (version 10.8) using data obtained from the FL1-H channel. Each strain was run in biological triplicate per condition on two separate occasions.

## References

[1] Boucher HW, Talbot GH, Bradley JS, Edwards JE, Gilbert D, Rice LB, Scheld M, Spellberg B, Bartlett J. 2009. Bad bugs, no drugs: no ESKAPE! An update from the Infectious Diseases Society of America. Clin Infect Dis 48:1–12. doi:10.1086/595011. 19035777

[2] Klevens RM, Morrison MA, Nadle J, Petit S, Gershman K, Ray S, Harrison LH, Lynfield R, Dumyati G, Townes JM, Craig AS, Zell ER, Fosheim GE, McDougal LK, Carey RB, Fridkin SK. Active Bacterial Core surveillance (ABCs) MRSA Investigators. 2007. Invasive methicillin-resistant *Staphylococcus aureus* infections in the United States. JAMA 298:1763–1771. doi:10.1001/jama.298.15.1763. 17940231

[3] Purrello S, Garau J, Giamarellos E, Mazzei T, Pea F, Soriano A, Stefani S. 2016. Methicillin-resistant *Staphylococcus aureus* infections: a review of the currently available treatment options. J Glob Antimicrob Resist 7:178–186. doi:10.1016/j.jgar.2016.07.010. 27889013

[4] Edwards B, Andini R, Esposito S, Grossi P, Lew D, Mazzei T, Novelli A, Soriano A, Gould I. 2014. Treatment options for methicillin-resistant *Staphylococcus aureus* (MRSA) infection: where are we now? J Glob Antimicrob Resist 2:133–140. doi:10.1016/j.jgar.2014.03.009. 27873719

[5] Ersoy SC, Abdelhady W, Li L, Chambers HF, Xiong YQ, Bayer AS. 2019. Bicarbonate resensitization of methicillin-resistant *Staphylococcus aureus* to β-lactam antibiotics. Antimicrob Agents Chemother 63:e00496-19. doi:10.1128/AAC.00496-19. 31010857 PMC6591647

[6] Ersoy SC, Heithoff DM, Barnes L, Tripp GK, House JK, Marth JD, Smith JW, Mahan MJ. 2017. Correcting a fundamental flaw in the paradigm for antimicrobial susceptibility testing. EBioMedicine 20:173–181. doi:10.1016/j.ebiom.2017.05.026. 28579300 PMC5478264

[7] Rose WE, Bienvenida AM, Xiong YQ, Chambers HF, Bayer AS, Ersoy SC. 2020. Ability of bicarbonate supplementation to sensitize selected methicillin-resistant *Staphylococcus aureus* (MRSA) strains to β-lactam antibiotics in an *ex vivo* simulated endocardial vegetation model. Antimicrob Agents Chemother 64:e02072-19. doi:10.1128/AAC.02072-19. 31844004 PMC7038310

[8] Ersoy SC, Otmishi M, Milan VT, Li L, Pak Y, Mediavilla J, Chen L, Kreiswirth B, Chambers HF, Proctor RA, Xiong YQ, Fowler VG, Bayer AS. 2020. Scope and predictive genetic/phenotypic signatures of ‘bicarbonate [NaHCO_3_]-responsiveness’ and β-lactam sensitization among methicillin-resistant *Staphylococcus aureus* (MRSA). Antimicrob Agents Chemother 64:e02445-19. doi:10.1128/AAC.02445-19. 32041719 PMC7179597

[9] Ersoy SC, Chambers HF, Proctor RA, Rosato AE, Mishra NN, Xiong YQ, Bayer AS. 2021. Impact of bicarbonate on PBP2a production, maturation, and functionality in methicillin-resistant *Staphylococcus aureus*. Antimicrob Agents Chemother 65:e02621-20. doi:10.1128/AAC.02621-20. 33649115 PMC8092911

[10] Ersoy SC, Hanson BM, Proctor RA, Arias CA, Tran TT, Chambers HF, Bayer AS. 2021. Impact of bicarbonate-β-lactam exposures on methicillin-resistant *Staphylococcus aureus* (MRSA) gene expression in bicarbonate-β-lactam-responsive vs. non-responsive strains. Genes 12:1650. doi:10.3390/genes12111650. 34828256 PMC8619011

[11] Ersoy SC, Rose WE, Patel R, Proctor RA, Chambers HF, Harrison EM, Pak Y, Bayer AS. 2021. A combined phenotypic-genotypic predictive algorithm for *in vitro* detection of bicarbonate: β-lactam sensitization among methicillin-resistant *Staphylococcus aureus* (MRSA). Antibiotics 10:1089. doi:10.3390/antibiotics10091089. 34572671 PMC8469475

[12] Ersoy SC, Chan LC, Yeaman MR, Chambers HF, Proctor RA, Ludwig KC, Schneider T, Manna AC, Cheung A, Bayer AS. 2022. Impacts of NaHCO_3_ on β-lactam binding to PBP2a protein variants associated with the NaHCO_3_-responsive versus NaHCO_3_-non-responsive phenotypes. Antibiotics 11:462. doi:10.3390/antibiotics11040462. 35453214 PMC9028190

[13] Ersoy SC, Manna AC, Proctor RA, Chambers HF, Harrison EM, Bayer AS, Cheung A. 2022. The NaHCO_3_-responsive phenotype in methicillin-resistant *Staphylococcus aureus* (MRSA) is influenced by *mecA* genotype. Antimicrob Agents Chemother 66:e00252-22. doi:10.1128/aac.00252-22. 35575577 PMC9211399

[14] Foster TJ. 2019. Can β-lactam antibiotics be resurrected to combat MRSA? Trends Microbiol 27:26–38. doi:10.1016/j.tim.2018.06.005. 30031590

[15] Brown S, Xia G, Luhachack LG, Campbell J, Meredith TC, Chen C, Winstel V, Gekeler C, Irazoqui JE, Peschel A, Walker S. 2012. Methicillin resistance in *Staphylococcus aureus* requires glycosylated wall teichoic acids. Proc Natl Acad Sci USA 109:18909–18914. doi:10.1073/pnas.1209126109. 23027967 PMC3503181

[16] Farha MA, Leung A, Sewell EW, D'Elia MA, Allison SE, Ejim L, Pereira PM, Pinho MG, Wright GD, Brown ED. 2013. Inhibition of WTA synthesis blocks the cooperative action of PBPs and sensitizes MRSA to β-lactams. ACS Chem Biol 8:226–233. doi:10.1021/cb300413m. 23062620 PMC3552485

[17] Campbell J, Singh AK, Santa Maria JP, Jr, Kim Y, Brown S, Swoboda JG, Mylonakis E, Wilkinson BJ, Walker S. 2011. Synthetic lethal compound combinations reveal a fundamental connection between wall teichoic acid and peptidoglycan biosyntheses in *Staphylococcus aureus*. ACS Chem Biol 6:106–116. doi:10.1021/cb100269f. 20961110 PMC3025082

[18] Maki H, Yamaguchi T, Murakami K. 1994. Cloning and characterization of a gene affecting the methicillin resistance level and the autolysis rate in *Staphylococcus aureus*. J Bacteriol 176:4993–5000. doi:10.1128/jb.176.16.4993-5000.1994. 8051012 PMC196337

[19] Winstel V, Xia G, Peschel A. 2014. Pathways and roles of wall teichoic acid glycosylation in *Staphylococcus aureus*. Int J Med Microbiol 304:215–221. doi:10.1016/j.ijmm.2013.10.009. 24365646

[20] Campbell J, Singh AK, Swoboda JG, Gilmore MS, Wilkinson BJ, Walker S. 2012. An antibiotic that inhibits a late step in wall teichoic acid biosynthesis induces the cell wall stress stimulon in *Staphylococcus aureus*. Antimicrob Agents Chemother 56:1810–1820. doi:10.1128/AAC.05938-11. 22290958 PMC3318382

[21] Swoboda JG, Campbell J, Meredith TC, Walker S. 2010. Wall teichoic acid function, biosynthesis, and inhibition. Chembiochem 11:35–45. doi:10.1002/cbic.200900557. 19899094 PMC2798926

[22] Sewell EW, Brown ED. 2014. Taking aim at wall teichoic acid synthesis: new biology and new leads for antibiotics. J Antibiot (Tokyo) 67:43–51. doi:10.1038/ja.2013.100. 24169797

[23] Xia G, Peschel A. 2008. Toward the pathway of *S. aureus* WTA biosynthesis. Chem Biol 15:95–96. doi:10.1016/j.chembiol.2008.02.005. 18291312

[24] Peschel A, Otto M, Jack RW, Kalbacher H, Jung G, Götz F. 1999. Inactivation of the *dlt* operon in *Staphylococcus aureus* confers sensitivity to defensins, protegrins, and other antimicrobial peptides. J Biol Chem 274:8405–8410. doi:10.1074/jbc.274.13.8405. 10085071

[25] Rahman MM, Hunter HN, Prova S, Verma V, Qamar A, Golemi-Kotra D. 2016. The *Staphylococcus aureus* methicillin resistance factor FmtA is a d-amino esterase that acts on teichoic acids. mBio 7:e02070-15–e02015. doi:10.1128/mBio.02070-15. 26861022 PMC4752606

[26] Qamar A, Golemi-Kotra D. 2012. Dual roles of FmtA in *Staphylococcus aureus* cell wall biosynthesis and autolysis. Antimicrob Agents Chemother 56:e00187-12. doi:10.1128/AAC.00187-12. PMC3393393 22564846

[27] Komatsuzawa H, Sugai M, Ohta K, Fujiwara T, Nakashima S, Suzuki J, Lee CY, Suginaka H. 1997. Cloning and characterization of the *fmt* gene which affects the methicillin resistance level and autolysis in the presence of Triton X-100 in methicillin-resistant *Staphylococcus aureus*. Antimicrob Agents Chemother 41:2355–2361. doi:10.1128/AAC.41.11.2355. 9371333 PMC164128

[28] Atilano ML, Pereira PM, Yates J, Reed P, Veiga H, Pinho MG, Filipe SR. 2010. Teichoic acids are temporal and spatial regulators of peptidoglycan cross-linking in *Staphylococcus aureus*. Proc Natl Acad Sci USA 107:18991–18996. doi:10.1073/pnas.1004304107. 20944066 PMC2973906

[29] Karinou E, Schuster CF, Pazos M, Vollmer W, Gründling A. 2019. Inactivation of the monofunctional peptidoglycan glycosyltransferase SgtB allows *Staphylococcus aureus* to survive in the absence of lipoteichoic acid. J Bacteriol 201:e00574-18. doi:10.1128/JB.00574-18. 30322854 PMC6287468

[30] Biswas R, Martinez RE, Göhring N, Schlag M, Josten M, Xia G, Hegler F, Gekeler C, Gleske A-K, Götz F, Sahl H-G, Kappler A, Peschel A. 2012. Proton-binding capacity of *Staphylococcus aureus* wall teichoic acid and its role in controlling autolysin activity. PLoS One 7:e41415. doi:10.1371/journal.pone.0041415. 22911791 PMC3402425

[31] Penyige A, Matkó J, Deák E, Bodnár A, Barabás G. 2002. Depolarization of the membrane potential by β-lactams as a signal to induce autolysis. Biochem Biophys Res Commun 290:1169–1175. doi:10.1006/bbrc.2001.6317. 11811985

[32] Chambers HF, Sachdeva M. 1990. Binding of β-lactam antibiotics to penicillin-binding proteins in methicillin-resistant *Staphylococcus aureus*. J Infect Dis 161:1170–1176. doi:10.1093/infdis/161.6.1170. 2345297

[33] Truesdell S, Zurenko G, Laborde A. 1989. Interaction of cephalosporins with penicillin-binding proteins of methicillin-resistant *Stapbylococcus aureus*. J Antimicrob Chemother 23:13–19. doi:10.1093/jac/23.suppl_D.13. 2722720

[34] Georgopapadakou NH, Liu FY. 1980. Binding of beta-lactam antibiotics to penicillin-binding proteins of *Staphylococcus aureus* and *Streptococcus faecalis*: relation to antibacterial activity. Antimicrob Agents Chemother 18:834–836. doi:10.1128/AAC.18.5.834. 6778388 PMC284100

[35] Schlag M, Biswas R, Krismer B, Kohler T, Zoll S, Yu W, Schwarz H, Peschel A, Götz F. 2010. Role of staphylococcal wall teichoic acid in targeting the major autolysin Atl. Mol Microbiol 75:864–873. doi:10.1111/j.1365-2958.2009.07007.x. 20105277

[36] Pereira SF, Henriques AO, Pinho MG, De Lencastre H, Tomasz A. 2009. Evidence for a dual role of PBP1 in the cell division and cell separation of *Staphylococcus aureus*. Mol Microbiol 72:895–904. doi:10.1111/j.1365-2958.2009.06687.x. 19400776 PMC2771448

[37] Turner RD, Ratcliffe EC, Wheeler R, Golestanian R, Hobbs JK, Foster SJ. 2010. Peptidoglycan architecture can specify division planes in *Staphylococcus aureus*. Nat Commun 1:1–9. doi:10.1038/ncomms1025. 20975691

[38] Rebets Y, Lupoli T, Qiao Y, Schirner K, Villet R, Hooper D, Kahne D, Walker S. 2014. Moenomycin resistance mutations in *Staphylococcus aureus* reduce peptidoglycan chain length and cause aberrant cell division. ACS Chem Biol 9:459–467. doi:10.1021/cb4006744. 24255971 PMC3944067

[39] Dorschner RA, Lopez-Garcia B, Peschel A, Kraus D, Morikawa K, Nizet V, Gallo RL. 2006. The mammalian ionic environment dictates microbial susceptibility to antimicrobial defense peptides. FASEB J 20:35–42. doi:10.1096/fj.05-4406com. 16394265

[40] Ridyard KE, Overhage J. 2021. The potential of human peptide LL-37 as an antimicrobial and anti-biofilm agent. Antibiotics 10:650. doi:10.3390/antibiotics10060650. 34072318 PMC8227053

[41] da Costa TM, de Oliveira CR, Chambers HF, Chatterjee SS. 2018. PBP4: a new perspective on *Staphylococcus aureus* β-lactam resistance. Microorganisms 6:57. doi:10.3390/microorganisms6030057. 29932109 PMC6164785

[42] Memmi G, Filipe SR, Pinho MG, Fu Z, Cheung A. 2008. *Staphylococcus aureus* PBP4 is essential for β-lactam resistance in community-acquired methicillin-resistant strains. Antimicrob Agents Chemother 52:3955–3966. doi:10.1128/AAC.00049-08. 18725435 PMC2573147

[43] Łeski TA, Tomasz A. 2005. Role of penicillin-binding protein 2 (PBP2) in the antibiotic susceptibility and cell wall cross-linking of *Staphylococcus aureus*: evidence for the cooperative functioning of PBP2, PBP4, and PBP2A. J Bacteriol 187:1815–1824. doi:10.1128/JB.187.5.1815-1824.2005. 15716453 PMC1064008

[44] García-Fernández E, Koch G, Wagner RM, Fekete A, Stengel ST, Schneider J, Mielich-Süss B, Geibel S, Markert SM, Stigloher C, Lopez D. 2017. Membrane microdomain disassembly inhibits MRSA antibiotic resistance. Cell 171:1354–1367.e20. doi:10.1016/j.cell.2017.10.012. 29103614 PMC5720476

[45] Romaniuk JA, Cegelski L. 2018. Peptidoglycan and teichoic acid levels and alterations in *Staphylococcus aureus* by cell-wall and whole-cell nuclear magnetic resonance. Biochemistry (Mosc) 57:3966–3975. doi:10.1021/acs.biochem.8b00495. PMC6309457 29806458

[46] Veiga H, Jorge AM, Pinho MG. 2011. Absence of nucleoid occlusion effector Noc impairs formation of orthogonal FtsZ rings during *Staphylococcus aureus* cell division. Mol Microbiol 80:1366–1380. doi:10.1111/j.1365-2958.2011.07651.x. 21477126

[47] Steele VR, Bottomley AL, Garcia-Lara J, Kasturiarachchi J, Foster SJ. 2011. Multiple essential roles for EzrA in cell division of *Staphylococcus aureus*. Mol Microbiol 80:542–555. doi:10.1111/j.1365-2958.2011.07591.x. 21401734

[48] Fenn WO. 1928. The carbon dioxide dissociation curve of nerve and muscle. Am J Physiol 85:207–223. doi:10.1152/ajplegacy.1928.85.2.207.

[49] Kahl BC, Goulian M, van Wamel W, Herrmann M, Simon SM, Kaplan G, Peters G, Cheung AL. 2000. *Staphylococcus aureus* RN6390 replicates and induces apoptosis in a pulmonary epithelial cell line. Infect Immun 68:5385–5392. doi:10.1128/IAI.68.9.5385-5392.2000. 10948168 PMC101802

[50] Monk IR, Tree JJ, Howden BP, Stinear TP, Foster TJ. 2015. Complete bypass of restriction systems for major *Staphylococcus aureus* lineages. mBio 6:e00308-15. doi:10.1128/mBio.00308-15. 26015493 PMC4447248

