# Supplementary material for: Influence of Sodium Bicarbonate on Wall Teichoic Acid Synthesis and β-Lactam Sensitization in NaHCO3-Responsive and Nonresponsive Methicillin-Resistant Staphylococcus aureus
Source: Microbiol Spectr. 2022 Nov 15;10(6):e03422-22. doi: 10.1128/spectrum.03422-22 (PMC9769754; doi:10.1128/spectrum.03422-22)
Supplement: Supplemental file 1 — Supplemental material. Download spectrum.03422-22-s0001.pdf, PDF file, 1.4 MB [file spectrum.03422-22-s0001.pdf]

Supplemental Figures and Tables

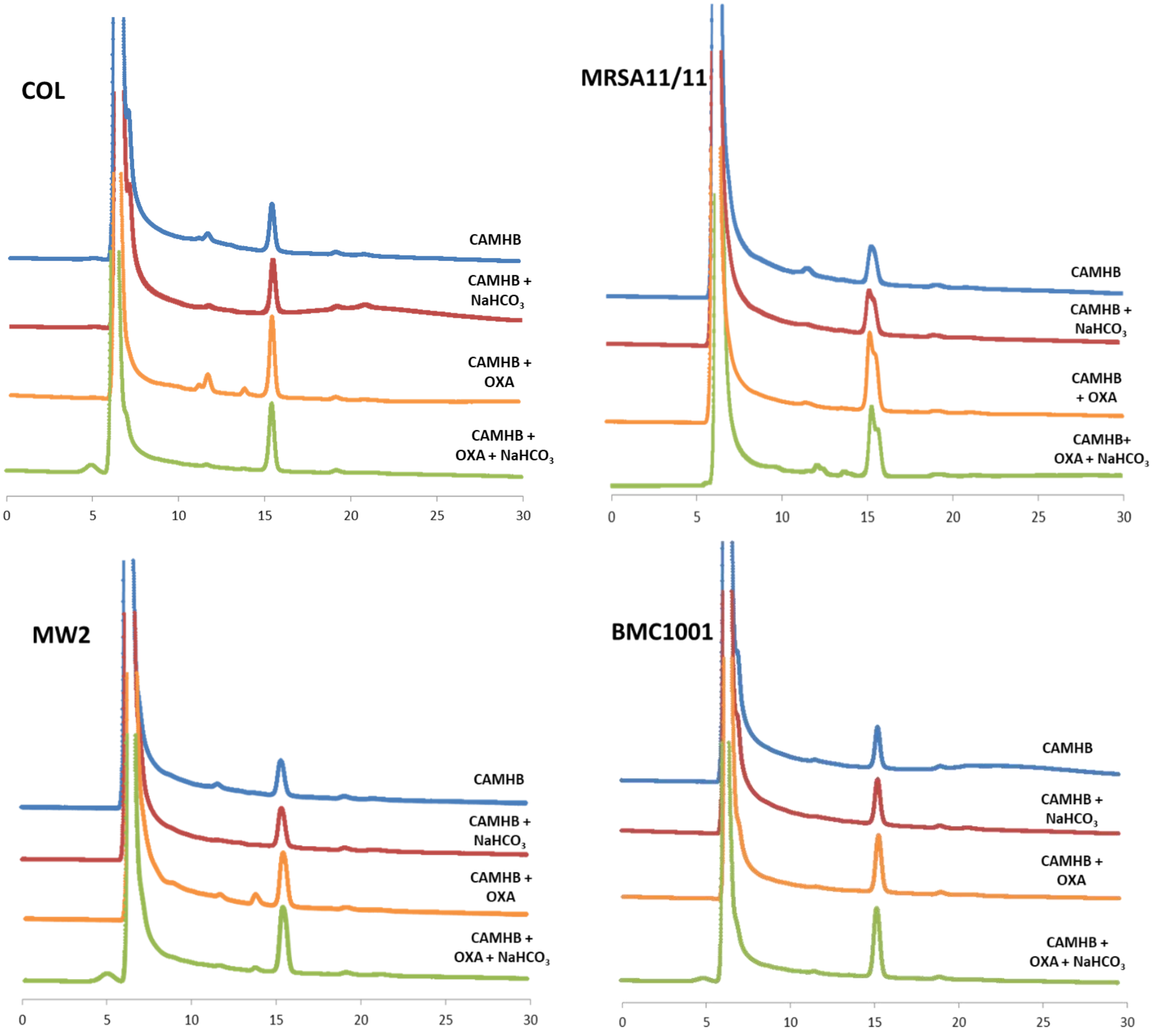

Figure S1. Determination of *O*-acetylation contents of *S. aureus* COL, BMC1001, MW2 and MRSA11/11 in CAMHB (blue); CAMHB + 44 mM $NaHCO_3$ (red); CAMHB + 2% NaCl+$^{1/2}$MIC oxacillin (128 µg/ml – COL; 64 µg/ml – BMC1001; 8 µg/ml- MW2 and MRSA11/11) (orange); CAMHB + 2% NaCl+$^{1/2}$MIC oxacillin + 44 mM $NaHCO_3$ (128 µg/ml – COL; 64 µg/ml – BMC1001; 0.25 µg/ml- MW2; 0.5 µg/ml -MRSA11/11) (green).

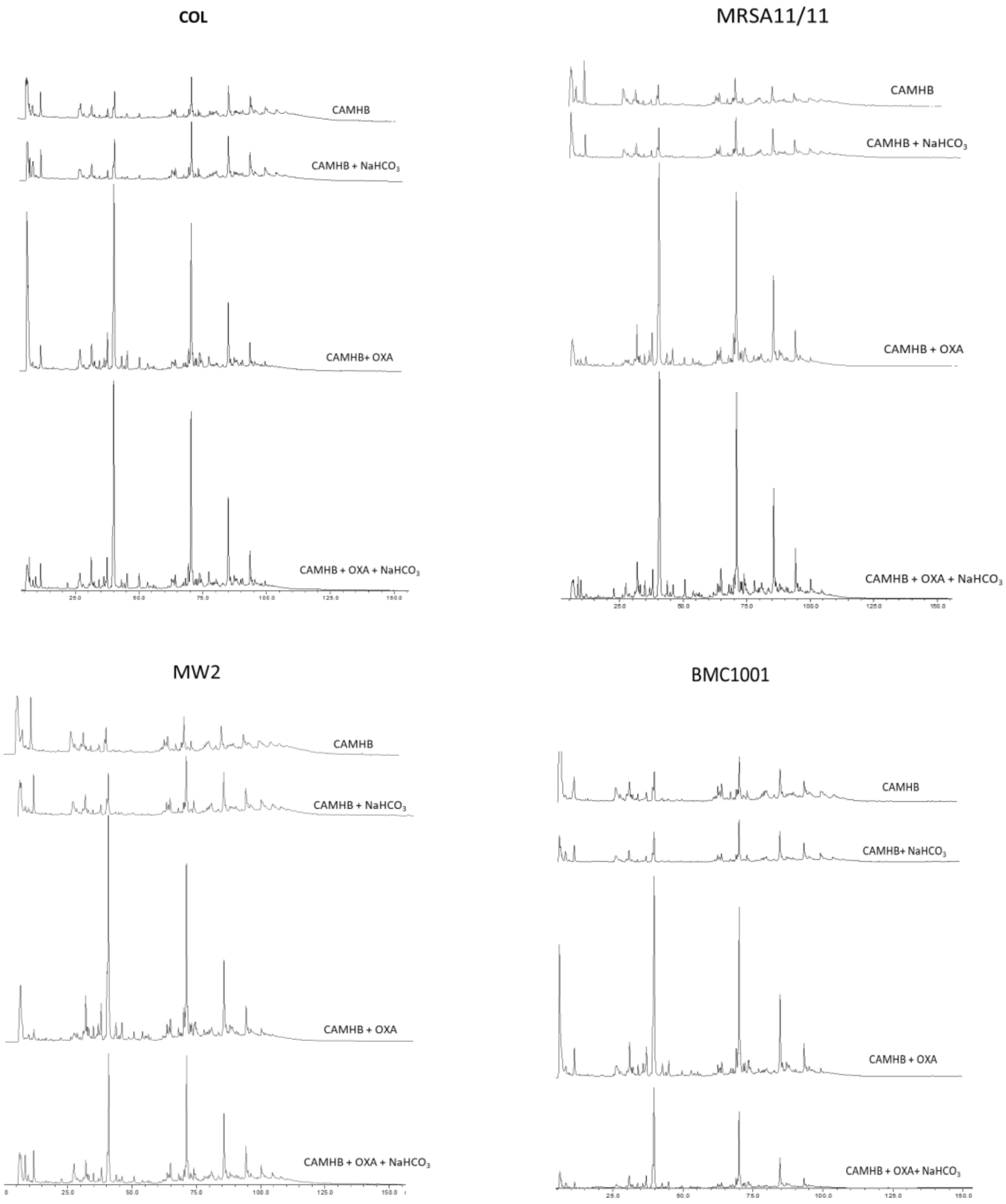

Figure S2. Peptidoglycan composition of *S. aureus* COL, BMC1001, MW2 and MRSA11/11 in CAMHB; CAMHB + 44 mM $NaHCO_3$; CAMHB +2% NaCl+$^{1/2}$MIC oxacillin (128 µg/ml – COL; 64 µg/ml – BMC1001; 0.5 µg/ml- MW2 and MRSA11/11); CAMHB +2% NaCl+$^{1/2}$MIC oxacillin + 44 mM $NaHCO_3$ (128 µg/ml – COL; 64 µg/ml – BMC1001; 0.5 µg/ml – MW2 and MRSA11/11).

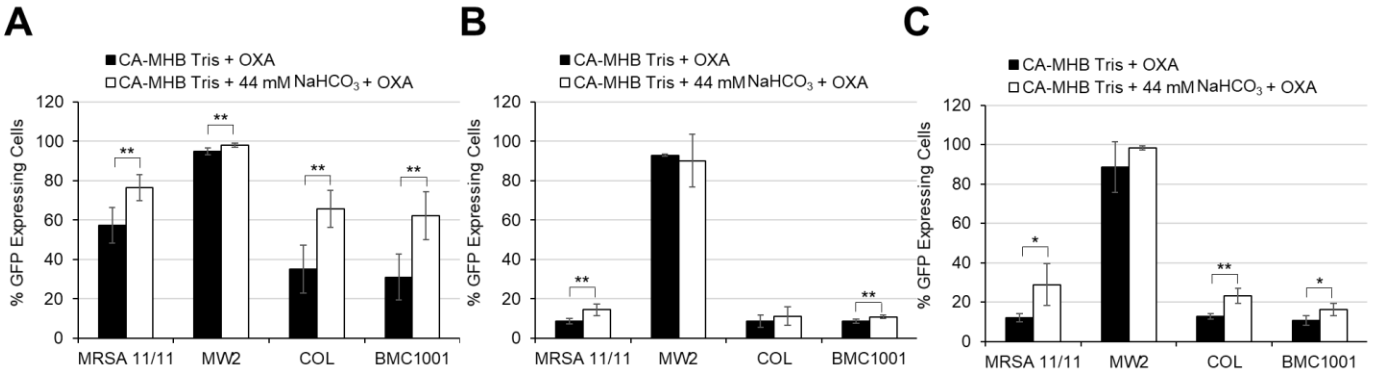

Figure S3. Flow cytometry to detect GFP expression in *tarO*, *tarG* and *dltA* translational fusions grown in the presence and absence of $NaHCO_3$, as measured by % cells expressing GFP in population of 10,000 cells.(A) *tarO*, (B) *tarG*, (C) *dltA*. Flow readings were taken after 6 h of incubation in the indicated condition. OXA exposures are equivalent to 1/2X the MIC in the indicated condition (MRSA 11/11: 16 µg/mL CA-MHB Tris, 0.25 µg/mL CA-MHB Tris + 44 mM $NaHCO_3$; MW2: 32 µg/mL CA-MHB Tris, 1 µg/mL CA-MHB Tris + 44 mM $NaHCO_3$; COL: 256 µg/mL both conditions; BMC1001: 128 µg/mL both conditions). Statistics calculated by a Student’s *t*-test, *P < 0.05, **P < 0.01.

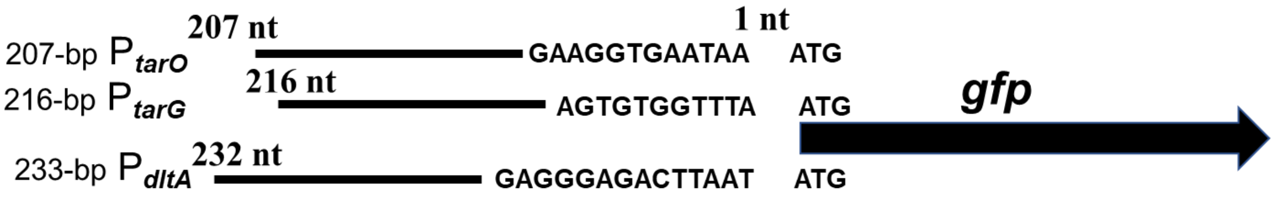

Figure S4. Schematic of translational constructs for *tarO*, *tarG*, and *dltA*. Numbers as indicated are marked based on the translation start codon of the respective genes.

Table S1. Acetic acid released (mg/L) by alkaline treatment of peptidoglycan of *S. aureus* strains COL, BMC1001, MW2 and MRSA11/11.

***S. aureus* strains**	**CAMHB**	**CAMHB + 44 mM NaHCO3**	**CAMHB + 2% NaCl + Oxacillin**	**CAMHB + 2% NaCl + Oxacillin + 44 mM NaHCO3**
COL	138.2	156.6	248.8	219.1
BMC1001	123.8	144.8	195.7	254.1
MW2	123.5	140.5	206.4	262.5
MRSA11/11	132.9	168.6	335.9	280.0

Table S2. Oxacillin minimum inhibitory concentrations (MICs, mg/L). In experiments in which sub-MIC concentrations of oxacillin were used, cells were exposed to 1/2 of the concentrations indicated in this table for each strain/condition.

Strain	CA-MHB	Ca-MHB 100 mM Tris	Ca-MHB 100 mM Tris 44 mM $NaHCO_3$
MRSA 11/11	16	32	0.5
MW2	16	64	2
COL	256	512	512
BMC1001	128	256	256

Table S3. qRT-PCR primers

Gene	Primer	Sequence (5' to 3')
tarO	Fwd	ACGTACTTGGGCTTGTAGATGA
	Rev	AGCAACTACTAAGGCAGCGG
tarG	Fwd	GGTGTGGCTTGGGAATTAAT
	Rev	CTTGGTTGATGAAGAACCAC
dltA	Fwd	GGCTTAACCAAGCGCCATTT
	Rev	TCGGTGTTGCTGTTAGCATT
fmtA	Fwd	TCATCGATTACAGACGAAGACACA
	Rev	AGTTTAGACGGCGCAACCTT
gyrB	Fwd	CGCAGGCGATTTTACCATTA
	Rev	GCTTTCGCTAGATCAAAGTCG

Table S4. Primers used for construction of *tarO*, *tarG*, and *dltA gfp* translational fusions.

Primer Name	Sequences (5' to 3')
tarO rbs F	AGGAGAATCTAGAGAAGGTGAATAAATGATTAAAGGAGAAGAACTTTTC ACT
tarG rbs F	AGGAGAATCTAGAAGTGTGGTTTAATGGAATGATTAAAGGAGAAGAA CTTTTCACT
dltA rbs F	AGGAGAATCTAGAGAGGGAGACTTAATATGATTAAAGGAGAAGAACTTTTCACT
1484 *EcoRI*	GGA TCC CCG GGT ACC GAG CTC
tarO EcoRI	AAG GAG GAA TTC GCC TTA AAT GAT ATG ATG AAA AAA TGA
tarO XbaI	AGGA GAA TCT AGA ATC GAT ATT AAT TGT TTT TAA CGT TAT A
tarG EcoRI	AAG GAG GAA TTC TGT TAA TCA TCA ATG ATA ACA ATG ATA
tarG XbaI	AGGA GAA TCT AGA TCA AAT GTT TAA CTA TTT CTC TAC TTA
dltA EcoRI	AAG GAG GAA TTC AAC GTA TTT TAT ATT GAA CGG TCT TTA
dltA XbaI	AGGA GAA TCT AGA CTC ATT AGA ACT CAT TAT AAA TGA AGT TA

Table S5. Translational fusion strains generated for this study.

Strain Number	Descriptions
ALC9423	MW2 (pALC1484 with 207-bp *tarO* promoter region with *tarO* ribosome binding site before *gfp* gene)
ALC9424	BMC1001 (pALC1484 with 207-bp *tarO* promoter region with *tarO* ribosome binding site before *gfp* gene)
ALC9425	MRSA11/11 (pALC1484 with 207-bp *tarO* promoter region with *tarO* ribosome binding site before *gfp* gene)
ALC9426	COL (pALC1484 with 207-bp *tarO* promoter region with *tarO* ribosome binding site before *gfp* gene)
ALC9427	MW2 (pALC1484 with 216-bp *tarG* promoter region with *tarO* ribosome binding site before *gfp* gene)
ALC9428	BMC1001 (pALC1484 with 216-bp *tarG* promoter region with *tarO* ribosome binding site before *gfp* gene)
ALC9429	MRSA 11/11 (pALC1484 with 216-bp *tarG* promoter region with *tarO* ribosome binding site before *gfp* gene)
ALC9430	COL (pALC1484 with 216-bp *tarG* promoter region with *tarO* ribosome binding site before *gfp* gene)
ALC9431	MW2 (pALC1484 with 232-bp *dltA* promoter region with *tarO* ribosome binding site before *gfp* gene)
ALC9432	BMC1001 (pALC1484 with 232-bp *dltA* promoter region with *tarO* ribosome binding site before *gfp* gene)
ALC9433	MRSA 11/11 (pALC1484 with 232-bp *dltA* promoter region with *tarO* ribosome binding site before *gfp* gene)
ALC9434	COL (pALC1484 with 232-bp *dltA* promoter region with *tarO* ribosome binding site before *gfp* gene)

Supplemental Methods

Isolation of peptidoglycan for O-acetylation determination (1). S. aureus COL, BMC1001, MRSA11/11 and MW2 were grown overnight in CA-MHB with and without $NaHCO_3$. These cultures were used to inoculate CA-MHB with and without $NaHCO_3$, and CA-MHB + 2% NaCl with $^{1/2}$MIC of oxacillin with and without $NaHCO_3$ to an initial OD= 0.0006. At OD=0.3 the cells were harvested by centrifugation, washed twice with 100 ml of cold 0.9% NaCl, resuspended in 20 ml of 0.9% NaCl and boiled in water for 20 min. After chilling on ice, the cells were centrifuged, washed twice with 40 ml of 0.9% NaCl, and resuspended in 2 ml of 0.9% NaCl. The cells were disrupted in a mechanical grinding device using glass beads, centrifuged, washed twice with 2 ml of cold dd-water, and boiled for 30 min in 10 ml of 2% SDS to remove non-covalently bound proteins. After centrifugation, the cell wall fragments were suspended in 2 ml of 0.1 M Tris-HCl (pH 6.8) and incubated with 0.5 mg/mL trypsin for 16 h at 37ºC with shaking to degrade cell-bound proteins. After centrifugation and washing with water, the cell walls were incubated for 5 h with 10% TCA to remove the teichoic acids. Purified peptidoglycan was isolated by centrifugation, washed four times with dd-water and lyophilized. To hydrolyze the *O*-acetyl groups, peptidoglycan (50 mg in 1.5 ml) was incubated for 3 h with 80 mM NaOH at 30°C with shaking. As a negative control, peptidoglycan was incubated for 3 h with 80 mM PBS (pH 6.4). The sample were centrifuged, and the released acetic acid was quantified by high-performance liquid chromatography (HPLC) on an organic acid column (HPX-87H; Bio-Rad) under the same conditions as the acetic acid standard (Bio-Rad) according to the instructions of the suppliers. Detection was performed at 205_{nm}. The samples were injected into the column and eluted with 0.008 M sulphuric acid at a flow rate of 0.6 ml.min^{-1} at 35°C.

Peptidoglycan purification. Cell wall isolation was performed as described previously (2, 3). *S. aureus* COL, BMC1001, MRSA11/11 and MW2 were grown overnight in CA-MHB with and without $NaHCO_3$. These cultures were used to inoculate CA-MHB with and without $NaHCO_3$ and

CA-MHB + 2% NaCl with $^{1/2}$MIC of oxacillin with and without antibiotic to an initial OD_{620}= 0.0006. At an OD_{620}=0.3, the cells were harvested by centrifugation, rapidly chilled in an ice-ethanol bath, and boiled in 4% SDS for 30 min. The cells were disrupted using 106-mm glass beads (**Sigma-Aldrich**) and subsequently treated with DNase (10 µg/ml; **Sigma-Aldrich**), RNase (50 µg/ml; **Sigma-Aldrich**), and trypsin (200 µg/ml; **Worthington Biochemical Corporation**, USA). Teichoic acids were removed by treatment with 49% hydrofluoric acid (**Merck**, Germany) for 48 h at 4°C. The peptidoglycan was lyophilized.

*Muropeptide analysis by RP-HPLC***.** Identical amounts of peptidoglycan of each sample were digested with mutanolysin (1 mg/ml; Sigma-Aldrich). This process was repeated in triplicate for each condition. The resulting muropeptides were reduced with sodium borohydride powder (**Sigma-Aldrich**) and separated by reverse-phase HPLC (RP-HPLC) using a Hypersil octyldecyl silane (**Runcorn**, Cheshire, UK) column and a linear gradient from 5% to 30% methanol in 100 mM sodium phosphate buffer, pH 2.5, at a flow rate of 0.5 ml/min as described previously (2, 3), using a Shimadzu Prominence system and Shimadzu LC solution software. The peaks of the chromatograms were identified by assessing the relative retention times according to previous studies (2, 3).

REFERENCES

1. Bera A, Herbert S, Jakob A, Vollmer W, Götz F. 2005. Why are pathogenic staphylococci so lysozyme resistant? The peptidoglycan O-acetyltransferase OatA is the major determinant for lysozyme resistance of Staphylococcus aureus. Mol Microbiol 55:778-787.
2. De Jonge B, Chang Y-S, Gage D, Tomasz A. 1992. Peptidoglycan composition of a highly methicillin-resistant Staphylococcus aureus strain. The role of penicillin binding protein 2A. J Biol Chem 267:11248-11254.
3. De Jonge B, Chang Y-S, Gage D, Tomasz A. 1992. Peptidoglycan composition in heterogeneous Tn551 mutants of a methicillin-resistant Staphylococcus aureus strain. J Biol Chem 267:11255-11259.
